# Dynamic monitoring of *UBA1* somatic mutations in patients with relapsing polychondritis

**DOI:** 10.1186/s13023-023-03003-x

**Published:** 2024-01-02

**Authors:** Suying Duan, Haiyang Luo, Yunchao Wang, Dongbin Jiang, Jiajia Liu, Jiaqi Li, Honglin Zheng, Taiqi Zhao, Chenyang Liu, Hang Zhang, Chengyuan Mao, Lei Zhang, Yuming Xu

**Affiliations:** 1 Department of Neurology, The First Affiliated Hospital of Zhengzhou University, Zhengzhou University, Zhengzhou, Henan China; 2 Rheumatology & Immunology Department, The First Affiliated Hospital of Zhengzhou University, Zhengzhou, China; 3 The Academy of Medical Sciences of Zhengzhou University, Zhengzhou University, Zhengzhou, Henan China; 4 Henan Key Laboratory of Cerebrovascular Diseases, The First Affiliated Hospital of Zhengzhou University, Zhengzhou University, Zhengzhou, Henan China

**Keywords:** Droplet digital PCR, *UBA1*, Somatic mutations, Relapsing polychondritis, Dynamic monitoring, VEXAS

## Abstract

**Background:**

Commonly clinically diagnosed with relapsing polychondritis (RP), vacuoles, E1 enzyme, X-linked, autoinflammatory, somatic syndrome (VEXAS) is a recently identified autoinflammatory disease caused by *UBA1* somatic mutations. The low frequency and dynamic changes challenge the accurate detection of somatic mutations. The present study monitored these mutations in Chinese patients with RP. We included 44 patients with RP. Sanger sequencing of *UBA1* was performed using genomic DNA from peripheral blood. Droplet digital polymerase chain reaction (ddPCR) was performed to screen low-prevalence somatic variants.

**Results:**

Multiple ddPCR detections were performed using available blood samples collected at different follow-up time points. Three male patients were *UBA1* somatic mutation carriers. Sanger sequencing detected the somatic *UBA1* variant c.122T > C (p.Met41Thr) in two male patients. Initial ddPCR confirmed the variant in the two patients, with allele fractions of 73.75% and 88.46%, respectively, while yielding negative results in other patients. Subsequent ddPCR detected the somatic variant (c.122T > C) with low prevalence (1.02%) in another male patient from blood samples collected at a different time point, and confirmed dynamically fractional abundance in one patient with VEXAS, with allele fractions of 73.75%, 61.28%, 65.01%, and 73.75%. Nine patients assessed by ddPCR at different time points remained negative.

**Conclusion:**

We report *UBA1* variants in patients with RP in the Chinese population for the first time. Multiple ddPCR detections from samples collected at different time points can enhance sensitivity and should be considered for patients with initial negative ddPCR results.

**Supplementary Information:**

The online version contains supplementary material available at 10.1186/s13023-023-03003-x.

## Background

Vacuoles, E1 enzyme, X-linked, autoinflammatory, somatic syndrome (VEXAS) is an adult-onset autoinflammatory disease caused by somatic mutations in *UBA1* (encoding ubiquitin-like modifier activating enzyme 1) [1, 2]. Prior to detecting *UBA1* somatic mutations in blood, the majority of patients with VEXAS are diagnosed with relapsing polychondritis (RP), as well as a small number with other inflammatory diseases, such as giant cell arteritis, polyarteritis nodosa, and Sweet syndrome [1]. RP is a rare inflammatory disease involving multiple systems throughout the body. Somatic mutation of *UBA1* gene is the genetic basis of VEXAS syndrome.

Sanger sequencing is a classic and practical method for gene variant screening in the targeted DNA fragment. However, Sanger sequencing cannot identify mutations representing less than 20% of total alleles, and low-prevalence somatic mutations are easily overlooked [3]. Droplet digital polymerase chain reaction (ddPCR) is a sensitive assay for determining a low-prevalence variant by quantifying the target DNA sequence. Somatic mutations are dynamic processes, and the dynamics challenge the accurate detection of somatic mutations by a single ddPCR assay. This study aimed to dynamically monitor these mutations in Chinese patients with RP by checking for *UBA1* somatic mutations by Sanger sequencing, whole-exome sequencing, and ddPCR, using available blood samples collected at different time points.

## Results

### Clinical characteristics of patients with RP

**Fig. 1:**
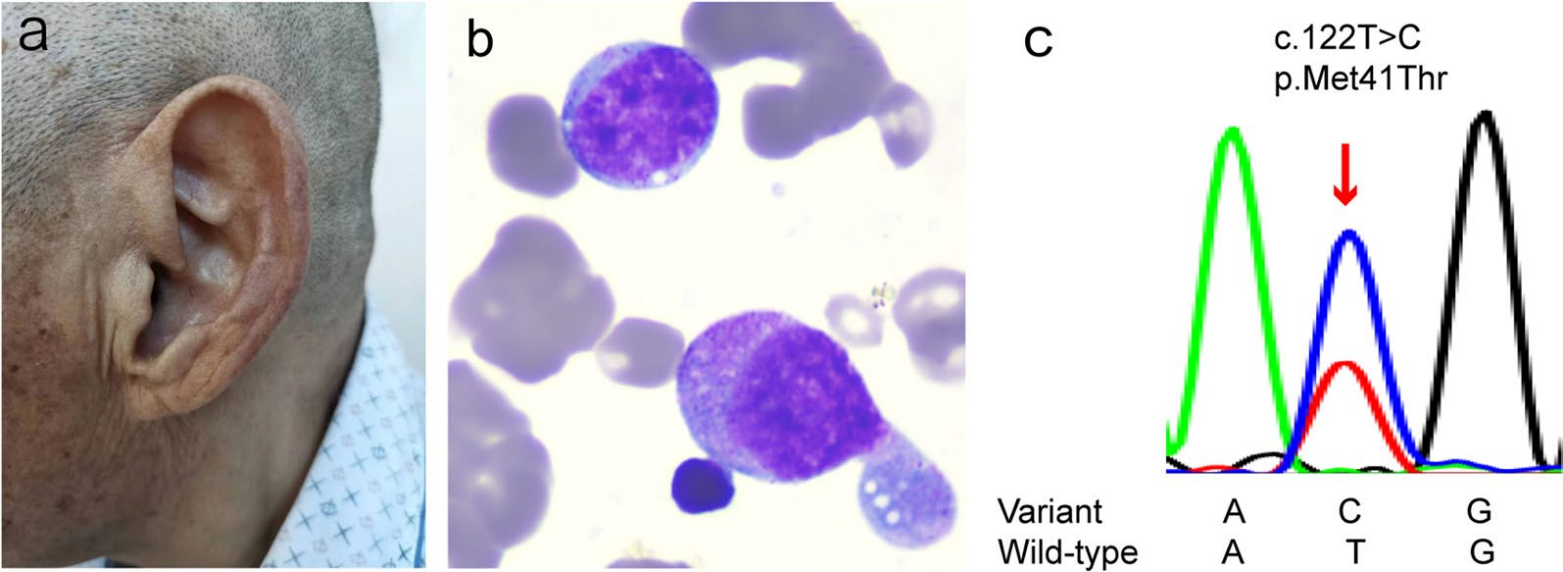
Clinical features of patient RP09 harboring a somatic variant in *UBA1*. **a** Auricular swelling. **b** Bone marrow aspirate showing vacuolization in myeloid and erythroid precursor cells (May–Giemsa staining, magnification, 40×). **c** Sanger sequencing map of somatic variation in *UBA1* in genomic DNA from peripheral blood

We included 44 patients with RP who met the diagnostic criteria in the present study. All participants were enrolled in research studies that had been approved by the institutional review board and provided written informed consent. The clinical characteristics of the 44 patients are summarized in Additional file 2. The onset age was high (mean ± SD, 50.34 ± 14.06 years), and 50% (22/44) of the patients were male. Among the patients, 61% (27/44) and 16% (7/44) presented with auricular and nasal chondritis, respectively. Other clinical features included eye inflammation (27%, 12/44), arthritis (23%, 10/44), airway involvement (50%, 22/44), fever (30%, 13/44), costal chondritis (14%, 6/44), skin rash (7%, 3/44), and cardiac involvement (9%, 4/44). The two patients with the *UBA1* somatic mutation identified by Sanger sequencing (RP09 and RP13) were older men with macrocytic anemia. No macrocytic anemia was found in the other 42 patients. It is worth noting that the third patient with a low VAF of *UBA1* (RP34) did not exhibit macrocytic anemia, but did have macrocytosis. We performed a bone marrow aspiration on patient RP09 and discovered vacuoles in myeloid and erythroid precursor cells (Fig. 1). Detailed clinical data on patients with somatic mutations of *UBA1* can be viewed in Additional file 5.

### Detection of somatic variants in *UBA1* by Sanger sequencing and Exome sequencing

Sanger sequencing for all the reported variants in *UBA1* was performed in 44 patients with RP. It identified the somatic variant c.122T > C (p.Met41Thr) in two male patients (RP09 and RP13) (Fig. 1). Exome sequencing of RP13 showed a 91% variant allele frequency subsequently confirmed by Sanger sequencing.

### Dynamically somatic *UBA1* mutations detected by multiple ddPCR

**Fig. 2:**
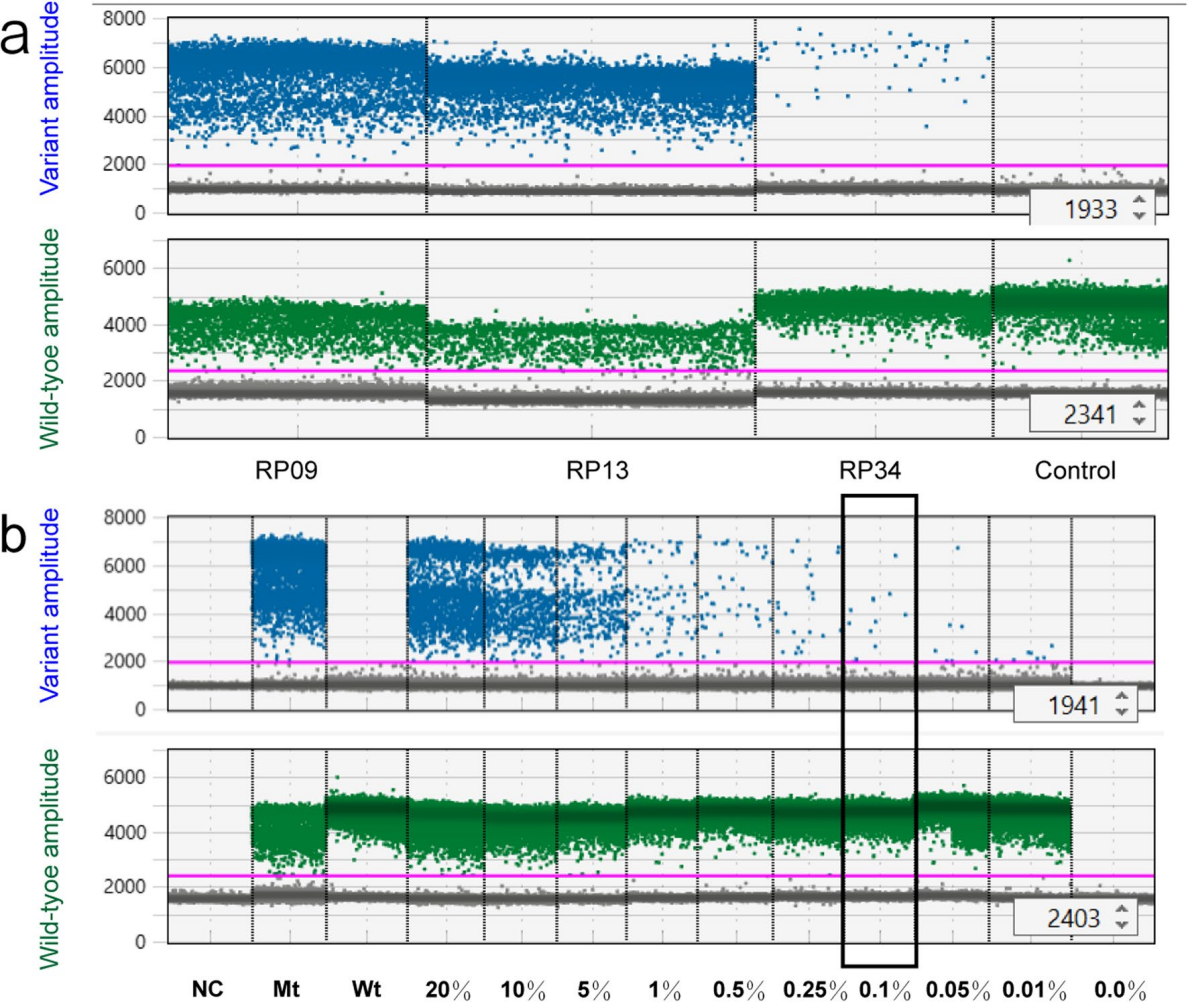
Droplet digital polymerase chain reaction detected *UBA1* variants in patients with RP. **a** A low-prevalence *UBA1* variant in patient RP34 was detected by droplet digital polymerase chain reaction (ddPCR) targeting c.122T > C. RP09 and RP13: variant-positive RP09 and RP13 (positive control), control: wild-type healthy (negative) control. Threshold amplitudes for ddPCR (pink lines) were > 1933 for the variant probe and > 2341 for the wild-type probe. Blue, green, and black dots represent variant (FAM-labelled), wild-type (HEX-labelled), and non-amplification signals, respectively. **b** The detection limit of ddPCR. Genomic DNA (gDNA) of a variant-positive patient (RP09, *UBA1* c.122T > C) was serially diluted with wild-type gDNA (control) to obtain variant allele ratios of 20.0%, 10.0%, 5.0%, 1.0%, 0.5%, 0.25%, 0.1%, 0.05%, and 0.0%. Samples with ratios of 20.0–0.1% were positively amplified by ddPCR

**Fig. 3:**
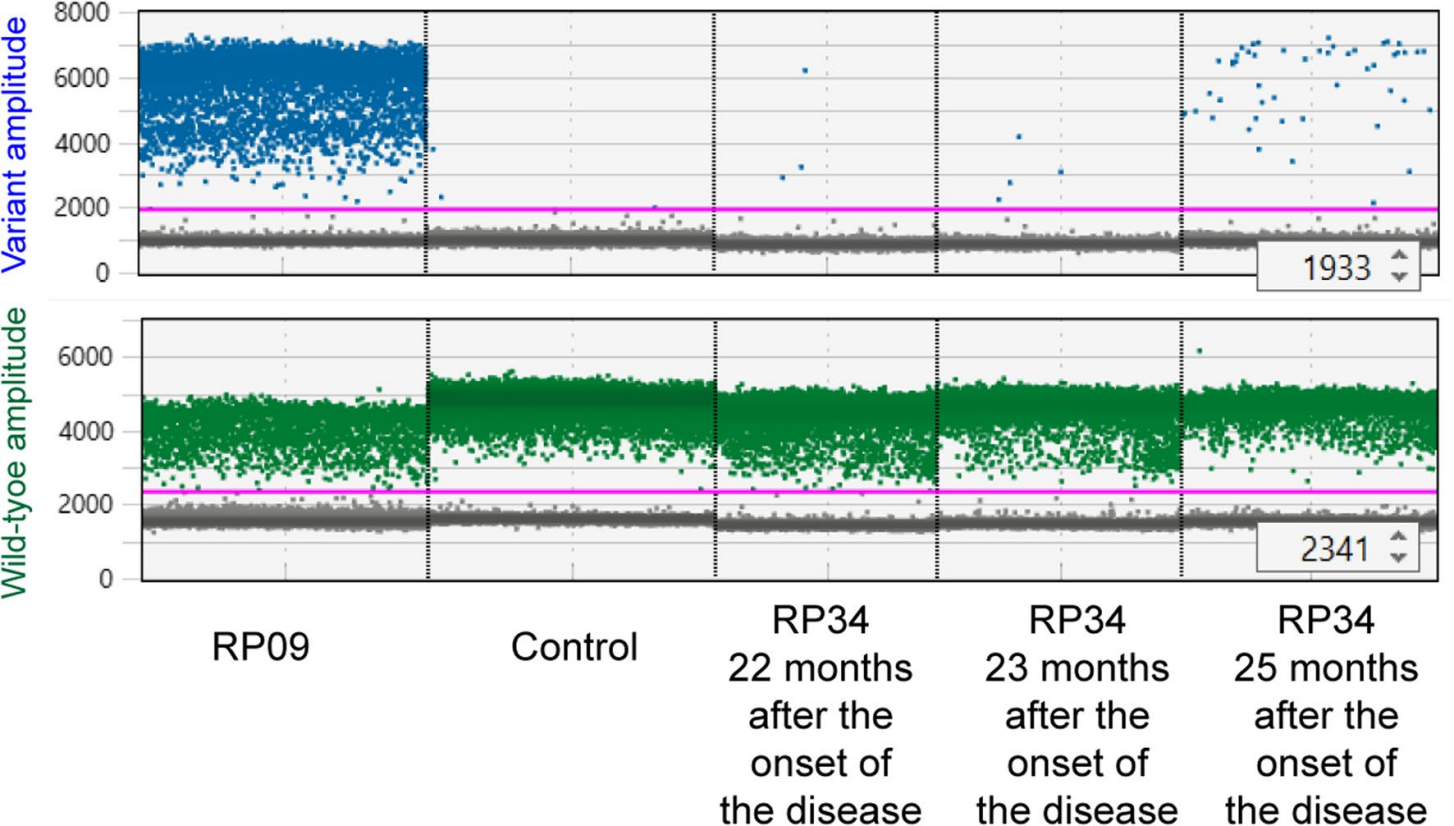
Multiple droplet digital polymerase chain reaction detected *UBA1* variants in samples collected at different time points from RP patients. Samples of patient RP34 were collected at three time points for droplet digital polymerase chain reaction (ddPCR). Only the ddPCR result of the last sample revealed a fractional abundance of 1.02% of c.121T > C

Single ddPCR detected the mutation in two patients, with variant allele frequencies of 73.75% and 88.46% (RP09 and RP13), while it yielded negative results in other patients. These are consistent with previous results from Sanger sequencing. Multiple ddPCR identified the somatic variant (c.122T > C) with low prevalence (1.02%) from another male patient (RP34) in one of all blood samples collected at different time points (Fig. 2a, Fig. 3, and Additional file 3). Peripheral blood of the patient was collected during his three visits to the hospital for re-examination on September 13, 2021, September 28, 2021, and November 16, 2021. The patient was not available for subsequent follow-up due to a change of residence.

An additional nine patients with RP (RP03, RP05, RP08, RP12, RP16, RP23, RP31, RP36, and RP40) were also sampled at different time points and assessed by ddPCR, with all results remaining negative (Additional file 3). The samples from patient RP09 were subjected to ddPCR detections of the *UBA1* variant at four different time points. The obtained fractional abundance was 73.75%, 61.28%, 65.01%, and 73.75%, confirming that somatic mutation was in dynamic change.

The detection limit of ddPCR was confirmed using serial dilutions of gDNA from a variant-positive patient (RP09, *UBA1* c.122T > C, 73.75% variant fractional abundance) along with control gDNA to obtain variant allele ratios of 20%, 10%, 5.0%, 1.0%, 0.5%, 0.25%, 0.1%, 0.05%, and 0.01%. The results confirmed that ddPCR reliably detected variant allele ratios of > 0.1% (predicted 15.7 copies) from 80 ng of gDNA per well (Fig. 2b and Additional file 3).

## Discussion

VEXAS (Vacuoles, E1 enzyme, X-linked, Autoinflammatory, Somatic) syndrome is a relatively recently identified condition linked to somatic mutations in the *UBA1* gene. Patients with VEXAS syndrome can present with a variety of clinical symptoms, including recurrent fevers, systemic inflammation, skin abnormalities (especially neutrophilic dermatoses), cytopenias, vacuoles in myeloid precursors, vascular thrombosis, pulmonary abnormalities and other systemic involvement. We conducted a case review analysis of three patients who tested positive for somatic mutations in the *UBA1* gene. We found that all three patients exhibited recurrent fevers, elevated inflammatory markers, erythema, swelling, and pain in the earlobes, and pneumonia. Among them, patients RP09 and RP13 also had macrocytic anemia, coagulation dysfunction, and neutrophilic skin disease. Vacuoles were observed in the myeloid precursor cells of patient RP09. Patient RP34 showed signs of macrocytosis.

To the best of our knowledge, we screened *UBA1* variants in Chinese patients with RP for the first time. One known pathogenic *UBA1* variant [1, 3, 4], c.122T > C (p.Met41Thr), was identified in 3 of the 44 patients (7%), consistent with a previously reported rate [5]. Thus, primary screening for *UBA1* somatic mutations in patients with RP by Sanger sequencing is feasible.

In the present study, Sanger sequencing identified two male patients with RP (RP09 and RP13) with somatic variants in *UBA1*. However, Sanger sequencing may miss low-level somatic mutations, especially those with less than 5% mutation frequency [6]. DdPCR is an effective method to detect low-level somatic mutations. For patient RP34, the results of Sanger sequencing for *UBA1* somatic mutations were negative, whereas subsequent ddPCR showed that the patient had a fractional abundance of 1.02% of the variant c.121T > C. Compared with traditional molecular methods, ddPCR has the advantages of high sensitivity and specificity, without the need for a standard curve for absolute quantification, good tolerance to PCR inhibitors, and high efficiency [7–10].

Previous studies have reported low frequency [11, 12] and dynamic changes [13, 14] in somatic variants. Therefore, to our knowledge, we performed multiple ddPCR detections on 11 patients with RP using blood samples collected at different follow-up time points for the first time. For samples collected from patient RP34 at three time points, the ddPCR result showed that the patient had a fractional abundance of 1.02%. The reason for this patient’s dynamic change in *UBA1* mutation rate is unclear; no similar studies have been reported. The observation may be related to changes in the patient’s condition. Previous studies have shown that the mutation rate of promyelocytic leukemia protein-retinoic acid receptor α decreases with improving the patient’s condition in patients with acute promyelocytic leukemia; the mutation rate will increase again in relapsing patients [15]. So we considered that the difference in three ddPCR results for RP34 patient was due to the changes in patients' conditions. In addition, we make two hypotheses: firstly, mutant cells may have the advantage of growth or survival, causing these cells to proliferate faster than normal cells. This can cause the frequency of mutated cells to increase over time. Secondly, the interaction of cells with their environment or random biological processes might also influence the frequency of mutated cells. Therefore, multiple ddPCR detections using samples collected at different time points should be considered for patients with negative single ddPCR results. Indeed, for patients who have already been diagnosed, multiple ddPCR tests may be unnecessary and could incur additional costs and inconvenience for the patients. There are two limitations to this study. On the one hand, the RP34 patient was lost to follow-up due to change of residence and was not able to perform another ddPCR to confirm the diagnosis; On the other hand, our study had a small sample size, and further research in a larger multicenter cohort is warranted to validate the improved sensitivity of multiple ddPCR.

In addition to ddPCR, two other techniques are currently used in VEXAS research: single-cell DNA sequencing and NGS. As VEXAS syndrome is caused by somatic mutations, single-cell DNA sequencing can be employed to study the distribution and abundance of these mutations in different cell populations or tissues. This aids in understanding the origin and development mechanisms of the disease. Gutierrez-Rodrigues et al. utilized single-cell DNA sequencing technology to uncover the spectrum of clonal hematopoiesis in VEXAS syndrome [16]. Through whole-genome or whole-exome sequencing, NGS enables researchers to quickly and comprehensively identify genetic variations associated with VEXAS syndrome, especially mutations in the *UBA1* gene. In our study, we performed whole-exome sequencing on the RP34 patient, which showed a 91% variant allele frequency. The ddPCR technique is particularly suitable for validating specific genetic variations identified in the results of NGS or single-cell DNA sequencing. Moreover, due to its high sensitivity, ddPCR can also be used to monitor the dynamic changes of somatic mutations, such as before and after treatment. In conclusion, single-cell DNA sequencing, NGS, and ddPCR each have their unique value in the study of VEXAS syndrome.

Ferrada et al. found that A decision tree algorithm based on male sex, a mean corpuscular volume > 100 fL, and a platelet count < 200 × 10^3^ /μL could differentiate VEXAS-RP from RP effectively [5]. Patients with RP09 and RP13 are all male, with a mean corpuscular volume > 100 fL and a platelet count < 200 × 10^3^ /μL. As shown by Ferrada et al., these biomarkers do predict *UBA1* mutation carrier status in RP patients. We reviewed all test results for patient RP34. This patient had undergone numerous complete blood count tests while in the hospital, and in two of them, we observed an elevated MCV while the platelet count remained normal. We speculate that this might be because the condition of patient RP34 is milder than that of patients RP09 and RP13, and there has not yet been any abnormality in the coagulation function.

## Conclusions

We identified a somatic *UBA1* variant in Chinese patients with RP for the first time. Multiple ddPCR detections are effective and feasible for detecting low-prevalence somatic mutations. These results indicate that conducting multiple ddPCR tests for the *UBA1* gene is significant in aiding the diagnosis of VEXAS syndrome for patients who are strongly suspected clinically of having VEXAS syndrome with initially negative *UBA1* mutations.

## Methods

### Study participants

We retrospectively collected the clinical information of patients with suspected RP admitted to the First Affiliated Hospital of Zhengzhou University between 2021 and 2022. Forty-four patients with confirmed RP (labeled RP01 to 44) were included in the study. Clinical diagnoses were defined according to standard criteria [17, 18]. Written informed consent was obtained from all participants included in the study following the Declaration of Helsinki. The authors affirm that human research participants provided informed consent for publication.

### DNA extraction and Sanger sequencing

Genomic DNA (gDNA) was extracted from peripheral blood leukocytes using a gDNA extraction kit (DP2102, BioTeke, Beijing, China) according to the manufacturer’s protocol. All gDNA samples were subjected to Sanger sequencing to screen for *UBA1* somatic mutations. All reported pathogenic variants, c.121A > C(p.Met41Leu), c.121A > G(p.Met41Val), c.122T > C(p.Met41Thr), c.167C > T(p.Ser56Phe), c.118-1G > C, c.118-2A > C and c.119-1G > C were identified using forward and reverse primers (see Additional file 1 for details).

### Multiple ddPCR targeting pathogenic *UBA1* variants

ddPCR was performed using a Droplet Digital PCR XQ200 system (Bio-Rad Laboratories, Hercules, CA, USA). Region-specific primers and customized locked nucleic acid probes for two wild-type *UBA1* (c.121A and c.122T) and three variant alleles (c.121A > C, c.121A > G, and c.122T > C) were purchased from Shandong Haichen Biotechnology Co. Ltd. (Shandong, China). The primers and probes are listed in Additional file 1. The PCR mixture contained 80 ng gDNA, 11 μL 2 × ddPCR Supermix for probes (no dUTP; Bio-Rad), 800 nM target-specific PCR primers, and 200 nM variant-specific and wild-type-specific locked nucleic acid probes. PCR mixture (20 μL) and Droplet Generation Oil for Probes (Bio-Rad) (70 μL) were mixed, and droplets were generated using a QX200 Droplet Generator (Bio-Rad) according to the manufacturer’s protocol. The droplet emulsion was subjected to thermal cycles as follows: denaturation at 95 °C for 10 min, 43 PCR cycles at 94 °C for 30 s and 52 °C for 1 min, and final extension at 98 °C for 10 min. PCR amplification in the droplets was confirmed using a QX200 Droplet Reader (Bio-Rad). The threshold was determined by comparing wild-type and no-template ddPCR results. All data above the threshold were evaluated. The data were analyzed using QX Manager (version 1.2, Bio-Rad).

### Confirmation of the ddPCR detection limit

Theoretically, 1 ng of gDNA should contain 330 copies. We found that 1 ng of gDNA from patient RP01 contained 242 copies of the variant *UBA1* allele, c.122T > C, according to the fractional abundance obtained by ddPCR. The gDNA of this patient was serially diluted with control wild-type gDNA in variant allele ratios of 20%, 10%, 5.0%, 1.0%, 0.5%, 0.1%, 0.05%, and 0.01%, and ddPCR was conducted on the serially diluted samples (using 80 ng of gDNA, equivalent to 2.64 × 10^4^ copies per well).

### Whole-exome sequencing

gDNA (1 mg) was randomly fragmented at Covaris LLC (Woburn, MA, USA). Fragmented DNA was selected by Agencourt AMPure XP-Medium kit (Beckman Coulter, Pasadena, CA, USA) to an average size of 150–250 bp. The selected fragments were subjected to end-repair, 3′-adenylation, adapter ligation, and PCR amplification. The PCR products were recovered using the AxyPrep Mag PCR Clean-up kit (Axygen, Union City, CA, USA). A certain amount of PCR products was used for hybridization using BGI hybridization (BGISEQ-500 platform, BGI, Shenzhen, China) and wash kits. Thereafter, the AxyPrep Mag PCR Clean-up kit was used to recover the products as described above. The double-stranded PCR products were heat-denatured and circularized. The splint oligo sequence forming the single-stranded circle DNA was used as the final library and qualified by quality control. The library was amplified to generate DNA nanoballs with more than 300 copies of one molecule. The DNA nanoballs were loaded into the patterned nanoarray, and pair-end 100-base reads were generated using combinatorial Probe-Anchor Synthesis on the BGISEQ-500 platform (BGI). The mean coverage depth against the RefSeq coding sequences was 136.57×, and ≥ 1 reads covered 99.89% of the coding sequences. Variants were confirmed by Sanger sequencing.

### Supplementary Information

**Additional file 1**. Sequences of primers and probes used in this study.

**Additional file 2**. Clinical features of patients with relapsing polychondritis and hematological abnormalities.

**Additional file 3.** Pathogenic variants detected by droplet digital PCR (ddPCR).

**Additional file 4**. Variant allele ratios detected by droplet digital PCR (ddPCR).

**Additional file 5**.Clinical features of patients with RP who had *UBA1* p.Met41 variants.

## Data Availability

All data generated or analysed during this study are included in this published article [and its supplementary information files].

## Abbreviations

ddPCR: Droplet digital polymerase chain reaction
gDNA: Genomic DNA
RP: Relapsing polychondritis
VEXAS: Vacuoles, E1 enzyme, X-linked, autoinflammatory, somatic syndrome
