# Supplementary material for: Dynamic monitoring of UBA1 somatic mutations in patients with relapsing polychondritis
Source: Orphanet J Rare Dis. 2024 Jan 2;19:1. doi: 10.1186/s13023-023-03003-x (PMC10762806; doi:10.1186/s13023-023-03003-x)
Supplement: Supplementary file 5 — Additional file 5.Clinical features of patients with RP who had UBA1 p.Met41 variants. [file 13023_2023_3003_MOESM5_ESM.pdf]

## Full case descriptions

### *Patient RP09*

The patient was a 71-year-old man who developed general fatigue and rashes on all limbs in 2020. Laboratory examination showed increased inflammatory markers, macrocellular anemia, and thrombocytopenia (Table E2). A chest CT scan showed interstitial changes in both lungs. Histopathologic evaluation of the bone marrow showed signs of vacuolization in myeloid and erythroid precursor cells.

### *Patient RP13*

The patient presented in 2021 at 64 years of age with fever, palpitations, chest tightness, and fatigue. Serologic analyses showed high inflammatory parameters and macrocytic anemia. The chest CT scan showed pneumonia. After admission, he developed hypotension and dyspnea. Septic shock was considered. Two months later, the patient suddenly showed a decrease in heart rate and died after rescue attempts.

### *Patient RP34*

The patient was a 45-year-old man who presented in 2019 with symptoms of fever, swelling and pain in the right ear. Laboratory examination showed increased inflammatory markers. The patient was treated with methylprednisolone (40 mg once a day), alfacalcidol (0.25 μg once a day), and loxoprofen sodium tablets (60 mg twice a day), and his symptoms subsequently improved.
